# Duração do Sono e Risco de Aterosclerose: Um Estudo Mendeliano de Randomização

**DOI:** 10.36660/abc.20230813

**Published:** 2024-08-21

**Authors:** Xiaozhuo Xu, Yilin Huang, Jing Liu, Xu Han

**Affiliations:** 1 Department of Geriatrics, Affiliated Hospital of Nanjing University of Chinese Medicine Nanjing China Department of Geriatrics, Affiliated Hospital of Nanjing University of Chinese Medicine, Jiangsu Province Hospital of Chinese Medicine, Nanjing – China

**Keywords:** Duração do Sono, Aterosclerose, Análise da Randomização Mendeliana

## Abstract

**Fundamento:**

A associação entre a duração do sono e a aterosclerose foi relatada em muitos estudos observacionais. No entanto, pouco se sabe sobre a sua importância como fator de risco para aterosclerose ou como consequência negativa da aterosclerose.

**Objetivo:**

Este estudo teve como objetivo avaliar a associação causal entre a duração do sono e o risco de aterosclerose usando estatísticas resumidas de estudos de associação genômica ampla (GWAS) disponíveis publicamente.

**Métodos:**

Empregamos um método de randomização mendeliana (RM) de duas amostras com 2 coortes do MRC-IEU (n = 460.099) e do UK Biobank (n = 361.194) para investigar a associação causal entre a duração do sono e o risco de aterosclerose. Três métodos, incluindo a técnica de variância inversa ponderada (IVW), escore de perfil ajustado robusto (RAPS) e abordagem de mediana simples e ponderada, foram usados para obter resultados confiáveis, e uma razão de chances com intervalo de confiança (IC) de 95% foi calculada. P<0,05 foi considerado diferença estatística. Além disso, foram utilizadas análises de regressão: MR-Egger regression, Radial MR, MR-PRESSO e leave-one-out para avaliar os possíveis efeitos de pleiotropia.

**Resultados:**

Não foi encontrada associação causal entre duração do sono e aterosclerose [OR (IC95%): 0,90 (0,98-1,00), p = 0,186]. As análises Leave-one-out, MR-Egger, e MR-PRESSO não conseguiram detectar pleiotropia horizontal.

**Conclusões:**

Esta análise de RM não indicou nenhuma associação causal entre a duração do sono geneticamente prevista e a aterosclerose nas populações europeias.

**Figure:**
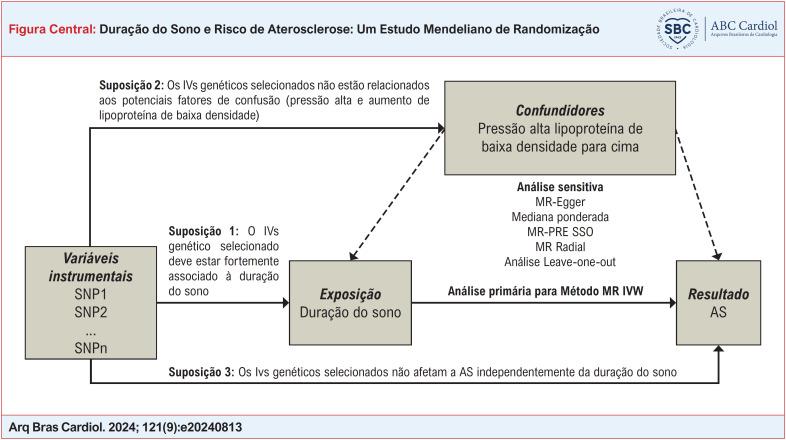


## Introdução

A aterosclerose é uma doença multifatorial e a principal causa de eventos cardiovasculares e cerebrovasculares.^1^ A aterosclerose é uma característica multifatorial complexa com uma etiologia genética enigmática. Sendo uma doença crónica que ameaça gravemente a saúde humana, tem despertado grande atenção, especialmente a aterosclerose coronária. Assistimos a uma "transição epidemiológica".^2^ O aumento do saneamento e o tratamento de infecções agudas reduziram a prevalência de doenças infecciosas nos países em desenvolvimento, e mais indivíduos sofrem agora de doenças crónicas, como a aterosclerose.^3^ A aterosclerose pode levar a uma variedade de doenças cardiovasculares (DCV), que têm sido reconhecidas como uma das principais causas de morbidade e mortalidade.^4^ É urgente interpretar o seu mecanismo, avançar na sua gestão e desenvolver perspectivas para mitigar o seu impacto.

O sono é um processo fisiológico complexo produzido pelo cérebro, que desempenha um papel muito importante na regulação das funções fisiológicas de vários sistemas do corpo. Com a contínua extensão do horário de trabalho na sociedade moderna, a forma como as pessoas trabalham e os hábitos de sono das pessoas também estão em constante mudança, e a redução do tempo de sono está a tornar-se um problema grave.^5^ Na verdade, descobriu-se que a duração curta e longa do sono está associada ao cálcio da artéria coronária^6-8^ e à espessura médio-intimal da carótida (CIMT),^9,10^ que são indicadores de aterosclerose em grandes artérias que alimentam o coração e o cérebro. Além disso, alguns estudos descobriram que um tempo de sono muito longo ou muito curto ainda aumentará a incidência de eventos cardiovasculares após o controle de fatores mistos, como obesidade, hipertensão e diabetes.^11^ Vários estudos observacionais também mencionaram a relação entre a duração do sono e a aterosclerose subclínica das artérias coronárias ou carótidas.^12,13^ No entanto, ainda não está claro se dormir pouco ou dormir demais contribui para a ocorrência de aterosclerose.

Até onde sabemos, a associação causal entre a duração do sono e a aterosclerose não foi avaliada. A randomização mendeliana (RM) é um método para verificar a causalidade, evitando confusão residual e superando a causalidade reversa em um cenário retrospectivo, que pode revelar estimativas causais de fatores de risco em doenças complexas usando variantes genéticas como variáveis instrumentais.^14,15^ Aqui, conduzimos um estudo de RM para avaliar a associação causal entre a duração do sono e o risco de aterosclerose.

## Métodos

### Design de estudo

Supondo que a estimativa causal dos estudos de RM seja credível. Três suposições cruciais precisam ser atendidas: 1) Deve haver uma forte associação entre as variáveis instrumentais genéticas (IVs) selecionadas e a exposição.^16^ 2) A escolha dos IVs genéticos não influencia o resultado sem considerar a exposição (ou seja, a pleiotropia horizontal é inexistente).^17^ 3) Os IVs genéticos selecionados não estão associados aos possíveis confundidores. A Figura Central fornece uma visão geral. Como a pesquisa foi baseada em conjuntos de dados acessíveis ao público e em estudos publicados anteriormente, a aprovação ética e o consentimento dos participantes não foram necessários para o estudo.

### Fontes de dados

As IVs para a duração do sono foram baseadas numa meta-análise de um estudo de associação genómica ampla (GWAS) de 460.099 pessoas de ascendência europeia. A duração habitual do sono autorreferida foi a principal exposição do presente estudo. Foi obtido a partir de questionários touchscreen na avaliação inicial. A duração do sono foi avaliada de acordo com uma pergunta padronizada: "Quantas horas você dorme a cada 24 horas?". Os participantes que responderam "Não sei" e "Prefiro não responder", e aqueles que forneceram durações de sono implausíveis (< 4 horas ou > 11 horas por dia) foram excluídos para minimizar a duração do sono implausível e potencial confusão por problemas de saúde. Uma descrição completa do desenho do estudo, dos participantes e dos métodos de controle de qualidade (CQ) foi descrita em detalhes anteriormente.^18^ O UK Biobank recebeu aprovação ética do Comitê de Ética em Pesquisa (a referência REC para o UK Biobank é 11/NW/0382).

A aterosclerose foi identificada com base nas 8ª e 10ª edições da Classificação Internacional de Doenças (CID). Os dados sobre aterosclerose foram coletados de participantes do United Kingdom Biobank (GWAS ID: ukb-d-I9_CORATHER, disponível em https://gwas.mrcieu.ac.uk/datasets/ukb-d-I9_CORATHER/). Este conjunto de dados incluiu 361.194 pessoas de ascendência europeia (um total de 14.334 casos e 346.860 controles) e incluiu 13.586.589 polimorfismos de nucleotídeo único (SNPs). Introduzimos a regressão de pontuação LD ajustada por covariável (cov-LDSC), um método para estimar com precisão a herdabilidade genética (h2g) e seu enriquecimento em populações homogêneas e misturadas com estatísticas resumidas e estimativas de LD na amostra. A divulgação completa dos dados continha a coorte de amostras genotipadas com sucesso (n=488.377). 49.979 indivíduos foram genotipados usando o arranjo UK BiLEVE e 438.398 usando o arranjo axioma UK Biobank. Um total de 9.851.867 SNPs de duração do sono em 460.099 indivíduos foram extraídos do MRC-IEU (GWAS ID: ukb-b-4424, disponível em https://gwas.mrcieu.ac.uk/datasets/ukb-b-4424/). CQ pré-imputação, faseamento e imputação foram conduzidos pelo estudo anterior.^19^

### A seleção das variáveis instrumentais relevantes

**Figura 1:**
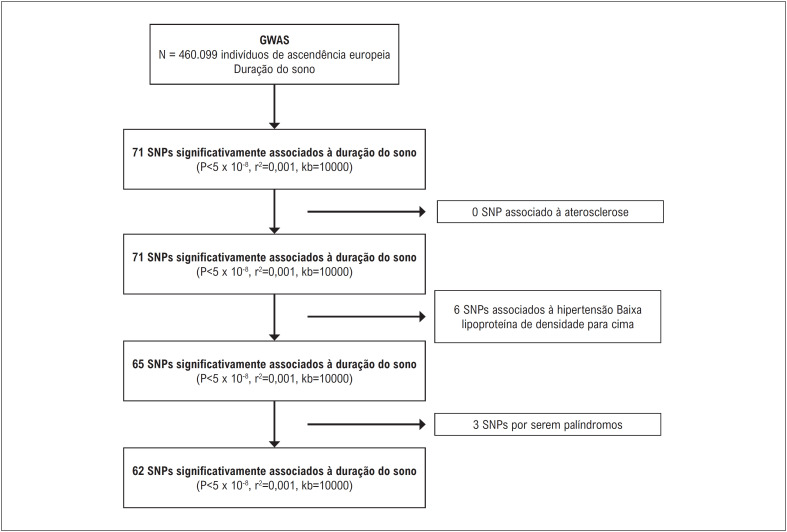
Fluxograma de seleção das variáveis instrumentais.

SNPs foram considerados IVs para este estudo.^16^ Os seguintes critérios foram satisfeitos por cada SNP solicitado: 1. Houve uma correlação substancial com a quantidade de exposição com base na relevância do genoma como um todo; 2. Sem desequilíbrio de ligação (LD) (r^2^ pareado = 0,001, tamanho da janela = 10.000kb); 3. Não contém estruturas palindrômicas. Um total de 65 SNPs foram encontrados após considerar as três suposições e critérios acima. Não conseguimos encontrar os SNPs apropriados no GWAS de aterosclerose, portanto, para obter estimativas precisas, empregamos SNPs proxy que tinham LD substancial (r^2^> 0,8) para substituir os SNPs escolhidos, o que nos permitiu obter resultados mais precisos. A regressão de primeiro estágio, ou estatística F, foi utilizada para avaliar a força dos instrumentos e foi calculada pela seguinte equação: F= (R^2^/k)/ ([1-R^2^]/[n-k-1]), onde R^2^ é a proporção da variabilidade da duração do sono contabilizada pelo SNP, k é o número de instrumentos utilizados no modelo e n é o tamanho da amostra.^20^ Para limitar a influência de um possível viés IV fraco, esperava-se que uma estatística F superior a 10 tivesse força suficiente para o estudo principal.^21^ O fluxograma para seleção de IVs está representado na Figura 1.

### Análise estatística

A abordagem ponderada pela variância inversa (IVW) foi utilizada como principal método para determinar se havia correlação entre a duração do sono e a aterosclerose.^22^ Se o p do teste Q de Cochran fosse superior a 0,05, optou-se por utilizar um modelo com efeitos fixos; em todos os outros casos, utilizamos um modelo com efeitos aleatórios.^23^ Se os IVs selecionados não apresentassem pleiotropia direcional (e o p para o intercepto Mr-Egger fosse maior que 0,05), a técnica IVW era considerada a mais confiável.^24^

Escolhemos a abordagem Mr-Egger para avaliar os possíveis impactos da pleiotropia nas análises de sensibilidade. O termo de interceptação da regressão Mr-Egger, que estimou o efeito causal como a inclinação da regressão ponderada das relações IVs-resultado na relação IVs-exposição, refletiu o efeito pleiotrópico médio.^25,26^ Para determinar se havia pleiotropia, também utilizamos as técnicas de teste de outlier de mediana básica, mediana ponderada, Radial MR e MR-PRESSO (Mendelian Randomization Pleiotropy Residual Sum and Outlier).^26^ Se mais de cinquenta por cento dos SNPs estudados fossem IVs eficazes, então a mediana ponderada oferecerá as estimativas mais confiáveis do impacto causal. Além da detecção de pleiotropia, o MR-PRESSO também pode reavaliar estimativas de efeitos e eliminar SNPs discrepantes.^26^ Para avaliar o impacto dos dados periféricos, foi entretanto realizada uma análise de exclusão. Investigamos ainda a pleiotropia de cada SNP escolhido usando o banco de dados PhenoScanner V2 (//www. pen scanner. medschl. cam. ac. uk/) no nível de significância estatística GWAS (p <5×10^-8^) para excluir o impacto de outras variáveis.^27^

Salvo indicação em contrário, todos os testes foram bilaterais e as diferenças foram consideradas estatisticamente significativas (p <0,05). Os pacotes Two Sample MR (V 0.5.6), Radial MR e MR-PRESSO (V 1.0)24 do software R foram usados para todas as análises estatísticas (4.0.5).

## Resultados

Mais informações sobre os SNPs escolhidos são fornecidas na Tabela Suplementar S1-S2. Três SNPs no total (duração do sono: rs1611719, rs17732997 e rs2186122) foram eliminados da pesquisa de RM porque eram palíndromos. No final, 62 SNPs, incluindo 1 SNP proxy, foram escolhidos como IVs (todos p < 5×10^-8^, r^2^=0,001).

### Estimativas de RM

Os resultados do teste Q de Cochran para o tempo de sono mostraram heterogeneidade mínima (p = 0,108). De acordo com os achados do método IVW, houve pouca evidência de ligação entre a duração do sono e o risco de aterosclerose (OR (IC 95%), 0,992 (0,979-1,004); p = 0,186) (Tabela Suplementar S3).

### Análises de sensibilidade

**Figura 2:**
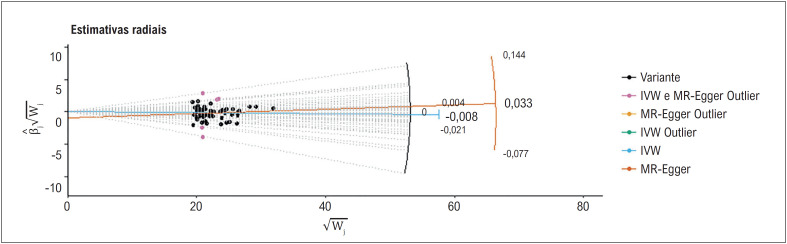
Uma visão geral do valor discrepante das estimativas radiais de RM.

Os resultados da mediana simples e da mediana ponderada foram comparáveis aos da abordagem IVW. Enquanto isso, a pleiotropia horizontal não foi detectada pela regressão MR-Egger (interceptação p = 0,071 para duração do sono) (Tabela Suplementar S3). Embora o MR Radial sugerisse a existência de outliers (Figura 2), o MR-PRESSO mostrou que os outliers não afetaram os resultados do estudo (Tabela 1). Da mesma forma, a pleiotropia não foi detectada pelo RAPS (Tabela 2) e pelo banco de dados PhenoScanner V2. Quando a pleiotropia horizontal apresentou p > 0,05, o método IVW (efeitos fixos) (Tabela 2) foi utilizado para avaliar os dados. Para a duração do sono, as Figuras Suplementares S1-S4 apresentam gráficos de floresta, gráficos de dispersão, gráficos de funil e gráficos de exclusão de RM.

**Tabela 1:** Estimativas MR-PRESSO entre duração do sono e aterosclerose

Característica	Estimativa bruta				Estimativa corrigida de outlier			
	N	OR	IC 95%	p	N	OR	IC 95%	p
Duração do sono	62	0,991	0,979-1,003	0,163	62	0,991	0,979-1,003	0,163

* SNP: polimorfismo de nucleotídeo único; MR-PRESSO: soma residual de pleiotropia de randomização mendeliana e teste de outlier. OR: razão de chances; IC95%: intervalo de confiança de 95%.

**Tabela 2:** Estimativas de RAPS e IVW (efeitos fixos) entre duração do sono e aterosclerose

Característica	RAPS				IVW (efeitos fixos)			
	N	OR	IC 95%	p	N	OR	IC 95%	p
Duração do sono	62	0,992	0,980-1,004	0,193	62	0,992	0,980-1,004	0,135

* SNP: polimorfismo de nucleotídeo único; RAPS: escore de perfil ajustado robusto; IVW (efeitos fixos), variância inversa ponderada (efeitos fixos). OR: razão de chances; IC: intervalo de confiança.

### Análises de viés e poder

O viés dos instrumentos genéticos foi de 0,000 para a duração do sono. A estatística F dos SNPs selecionados foi de 15.671, o que se esperava ter força suficiente para o estudo principal (Tabela Suplementar S3). A estimativa derivada da técnica de razão foi próxima da razão de chances condicional sob certas condições particulares e se aproximou de uma razão de chances média populacional.^28,29^ A consistência do estimador sob o valor nulo não foi afetada pela estimativa do odds ratio utilizada. Realizamos os cálculos de potência e o valor do erro Tipo 1 para a duração do sono foi de 0,05. Para o valor do poder estatístico do sono, a duração foi de 95%. De acordo com o tamanho da amostra utilizado na meta-análise de aterosclerose GWAS, houve poder> 80% para identificar a relação entre a quantidade de sono e o risco de aterosclerose para tamanho de efeito (OR) de 0,992 (Tabela Suplementar S4). Em recentes investigações adicionais de RM, todos os IVs para a duração do sono geneticamente prevista foram autorizados e utilizados.^30,31^ Além disso, nenhum deles teve qualquer influência na hipertensão arterial ou níveis elevados de lipoproteína de baixa densidade (Tabela Suplementar S3).

## Discussão

No presente estudo, tentamos explorar a associação causal entre duração do sono e aterosclerose usando um método de RM. As nossas descobertas não mostraram nenhuma evidência de que a duração do sono geneticamente prevista esteja ligada ao risco de aterosclerose nas populações europeias. Além disso, estudos de sensibilidade mostraram que os resultados eram geralmente confiáveis.

Os escores de cálcio arterial coronariano (CACS), CIMT e velocidade da onda de pulso braquial-tornozelo (baPWV) foram os principais indicadores substitutos de aterosclerose e preditores de eventos cardiovasculares.^6,32^ Alguns estudos exploraram o efeito da duração do sono na incidência de aterosclerose, analisando a relação entre a duração do sono e CACS, CIMT e baPWV. Um estudo recente com 1.968 homens saudáveis com idades entre 40 e 60 anos indicou que o aumento ou diminuição da duração do sono estava associado a um aumento na incidência de aterosclerose coronariana e avaliou o efeito da duração do sono na incidência de arteriosclerose subclínica medindo a CACS, encontrando pessoas que dormiam por 7 horas teve a menor incidência de aterosclerose coronariana subclínica.^33^ Para pessoas com fatores de risco para aterosclerose, a duração do sono também foi significativamente correlacionada com a incidência de aterosclerose. Da mesma forma, a CIMT foi mais baixa quando o tempo de sono foi de 7 a 8 horas, e o aumento ou diminuição do tempo de sono levará a um aumento da CIMT.^12^

Estudos anteriores não mostraram relação entre a duração do sono e marcadores de dano vascular e aterosclerose.^9,34-38^ Uma pesquisa transversal com 1.093 homens japoneses relatou que a duração do sono autorreferida não estava associada ao aumento da CAC ou da CIMT.^33^ Souza et al.^35^ também não encontraram associações independentes da duração objetiva do sono com a CIMT. Nenhuma evidência demonstrou que a associação entre sintomas de insônia e pontuação CAC> 0 diferiu de acordo com o status objetivo de curta duração do sono.^36^ Além disso, um estudo sobre o Estudo Multiétnico de Aterosclerose (MESA) mostrou que a apneia obstrutiva do sono grave não estava associada a alta carga de CAC ou ITB anormal.^37^ Os investigadores do MESA não relataram associações entre durações de sono curtas (<6 horas) e longas (>8 horas) e CAC.^38^ Estes foram consistentes com nossas descobertas.

A ligação clínica e fisiologicamente significativa entre o sono prolongado e o risco de DCV em adultos não é apoiada por dados experimentais suficientes. Nossa hipótese, com base nas informações atuais, é que o mecanismo subjacente era de natureza metabólica e operava por via inflamatória. Especificamente, o sono prolongado pode levar a níveis baixos de HDL,^39,40^ hiperglicemia, hipertrigliceridemia^41^ e resistência à insulina,^42^ os quais podem levar à disfunção endotelial vascular e à inflamação subclínica, promovendo ainda mais a aterosclerose.^43,44^ Questões sociais, de estilo de vida e comportamentais relacionadas, como o abuso de drogas, a inatividade física ou a falta de acesso a refeições nutritivas, podem exacerbar este ambiente pró-aterogénico.^45,46^ Independentemente da real relação de causa e efeito, apoiamos o exame da duração do sono nas avaliações clínicas, uma vez que a duração curta ou longa do sono pode indicar risco de doenças crônicas. As DCV e a diabetes são doenças potencialmente fatais que prevalecem na nossa sociedade e podem levar à doença precoce e à morte, pelo que é importante investigar a relação entre o sono e as doenças crónicas ao longo do tempo. Isto inclui encontrar a melhor estratégia de prevenção para alertar contra a aterosclerose, que pode estimular DCV e outras doenças. Este é um passo importante para uma população mais saudável, tanto a nível nacional como global.

De acordo com o nosso conhecimento, este estudo foi a primeira investigação de RM a examinar a associação causal entre a duração do sono e o risco de aterosclerose utilizando conjuntos de dados GWAS disponíveis. Além disso, para esta investigação de RM de duas amostras, escolhemos pessoas da Europa para diminuir o preconceito demográfico. A atual pesquisa de RM também apresentou várias deficiências. Primeiro, como utilizamos dados genéticos acessíveis ao público para a nossa investigação, não fomos capazes de fazer análises estratificadas ou levar em consideração fatores adicionais. Em segundo lugar, os SNPs instrumentais escolhidos como IVs explicaram apenas parcialmente (0,001% -0,01%) a variação na duração do sono. Isto pode resultar de um baixo poder estatístico para identificar relações fracas. Terceiro, numa análise de RM de duas amostras, qualquer viés devido a instrumentos fracos foi na direção do nulo. O viés na direção do nulo foi menos sério do que o viés na direção da associação observacional, pois é conservador e não levará a taxas de erro tipo 1 inflacionadas e a resultados falso-positivos. Havia de fato uma possibilidade de sobreposição entre as duas amostras.^28^ Em última análise, uma vez que o nosso conjunto de dados era composto por pessoas de herança europeia, as nossas conclusões podem não se aplicar a outros grupos fora da Europa.

## Conclusões

No presente estudo, a duração do sono geneticamente prevista entre as populações europeias não estava causalmente ligada ao risco de aterosclerose. Mais estudos são necessários para investigar a associação causal entre aterosclerose e duração do sono.

## *Material suplementar

Para informação adicional da Tabela Suplementar, por favor, clique aqui.

Para informação adicional da Figura Suplementar, por favor, clique aqui.
