# Supplementary material for: Duração do Sono e Risco de Aterosclerose: Um Estudo Mendeliano de Randomização
Source: Arq Bras Cardiol. 2024 Aug 21;121(9):e20240813. [Article in Portuguese] doi: 10.36660/abc.20230813 (PMC11495613; doi:10.36660/abc.20230813)
Supplement: Translation

# Translation: Sleep Duration and the Risk of Atherosclerosis: A Mendelian Randomization Study

**DOI:** 10.36660/abc.20230813i

**Authors:** Xiaozhuo Xu, Yilin Huang, Jing Liu, Xu Han

**Affiliations:** 1 Department of Geriatrics, Affiliated Hospital of Nanjing University of Chinese Medicine Nanjing China Department of Geriatrics, Affiliated Hospital of Nanjing University of Chinese Medicine, Jiangsu Province Hospital of Chinese Medicine, Nanjing – China

**Keywords:** Sleep Duration, Atherosclerosis, Mendelian Randomization Analysis

## Abstract

**Background:**

The association between the length of sleep and atherosclerosis has been reported in many observational studies. However, little is known about its significance as a risk factor for atherosclerosis or as a negative consequence of atherosclerosis.

**Objective:**

This study aimed to assess the causal association between sleep duration and the risk of atherosclerosis using publicly available genome-wide association studies (GWAS) summary statistics.

**Methods:**

We employed a two-sample Mendelian randomization (MR) method with 2 cohorts from MRC-IEU (n=460,099) and UK Biobank (n=361,194) to investigate the causal association between sleep duration and the risk of atherosclerosis. Three methods including the inverse-variance weighted (IVW) technique, Robust adjusted profile score (RAPS), and simple-and weighted-median approach were used to obtain reliable results, and an odds ratio with a 95% confidence interval (CI) was calculated. P<0.05 was considered as a statistical difference. In addition, MR-Egger regression, Radial MR, MR-PRESSO, and leave-one-out analyses were used to assess the possible pleiotropy effects.

**Results:**

No causal association of sleep duration with atherosclerosis was found [OR (95%CI): 0.90 (0.98-1.00), p = 0.186]. Leave-one-out, MR-Egger, and MR-PRESSO analyses failed to detect horizontal pleiotropy.

**Conclusions:**

This MR analysis indicated no causal association between genetically predicted sleep duration and atherosclerosis across European populations.

## Introduction

Atherosclerosis is a multifactorial disease and the leading cause of cardiovascular and cerebrovascular events.^1^ Atherosclerosis is a complex multifactorial trait with an enigmatic genetic etiology. As a chronic disease severely threatening human health, it has aroused wide attention, especially coronary atherosclerosis. We have witnessed an "epidemiological transition".^2^ Increased sanitation and the treatment of acute infections have reduced the prevalence of infectious diseases in developing countries, and more individuals are now experiencing chronic diseases such as atherosclerosis.^3^ Atherosclerosis can lead to a variety of cardiovascular diseases (CVDs), which have been recognized as a major cause of morbidity and mortality.^4^ It is urgent to interpret its mechanism, advance its management, and develop prospects for mitigating its impact.

Sleep is a complex physiological process produced by the brain, which plays a very important role in regulating the physiological functions of various systems in the body. With the continuous extension of working hours in modern society, the way people work and the sleep habits of people are also constantly changing, and the reduction of sleep time is becoming a severe problem.^5^ In fact, short and long sleep duration was found to be associated with coronary artery calcium^6-8^ and carotid intima-media thickness (CIMT),^9,10^ which are indicators of atherosclerosis in large arteries feeding the heart and brain. In addition, some studies have found that too long or too short sleep time will still increase the incidence of cardiovascular events after controlling for mixed factors such as obesity, hypertension, and diabetes.^11^ Several observational studies have also mentioned the relationship between sleep duration and subclinical atherosclerosis of coronary or carotid arteries.^12,13^ However, it remains unclear whether not getting enough or too much sleep contributes to the occurrence of atherosclerosis.

To the best of our knowledge, the causal association between sleep duration and atherosclerosis has not been assessed. Mendelian randomization (MR) is a method for verifying the causality, avoiding residual confounding, and overcoming reverse causality in a retrospective setting, which can reveal causal estimates of risk factors in complex diseases using genetic variants as instrumental variables.^14,15^ Herein, we conducted an MR study to evaluate the causal association between sleep duration and the risk of atherosclerosis.

## Methods

### Study design

Assuming the MR studies’ causal estimate is credible. Three crucial assumptions need to be met: 1) There must be a strong association between the selected genetic instrumental variables (IVs) and exposure.^16^ 2) The choice of genetic IVs does not influence the outcome without consideration of exposure (i.e., horizontal pleiotropy is nonexistent).^17^ 3) The selected genetic IVs are not associated with the possible confounders. Central Illustration provides an overview. Since the research was based on publicly accessible datasets and previously published studies, ethical approval and participant consent were not required for the study.

### Data sources

IVs for sleep duration were based on a meta-analysis of a genome-wide association study (GWAS) of 460,099 people of European ancestry. Self-reported habitual sleep duration was the major exposure of the current study. It was obtained from touchscreen questionnaires at baseline assessment. Sleep duration was evaluated according to a standardized question: "How many hours of sleep do you get every 24 hours?". Participants who answered "Do not know" and "Prefer not to answer", and those who provided implausible sleep durations (< 4 h or > 11 h per day) were excluded to minimize implausible sleep duration and potential confounding by poor health. A complete description of the study design, participants, and quality control (QC) methods has been described in detail previously.^18^ UK Biobank received ethical approval from the Research Ethics Committee (REC reference for UK Biobank is 11/NW/0382).

Atherosclerosis was identified based on the 8^th^ and 10^th^ editions of the International Classification of Diseases (ICD). Data on atherosclerosis were collected from participants in the United Kingdom Biobank (GWAS ID: ukb-d-I9_CORATHER, available at https://gwas.mrcieu.ac.uk/datasets/ukb-d-I9_CORATHER/). This data set included 361,194 people of European ancestry (a total of 14,334 cases and 346,860 controls), and it included 13,586,589 single nucleotide polymorphisms (SNPs). We introduced covariate-adjusted LD score regression (cov-LDSC), a method to accurately estimate genetic heritability (h^2^g) and its enrichment in both homogenous and admixed populations with summary statistics and in-sample LD estimates. The full data release contained the cohort of successfully genotyped samples (n=488,377). 49,979 individuals were genotyped using the UK BiLEVE array and 438,398 using the UK Biobank axiom array. Totally 9,851,867 SNPs of sleep duration in 460,099 individuals were extracted from the MRC-IEU (GWAS ID: ukb-b-4424, available at https://gwas.mrcieu.ac.uk/datasets/ukb-b-4424/). Pre-imputation QC, phasing, and imputation were conducted by the previous study.^19^

### The selection of the relevant instrumental variables

SNPs were considered as IVs for this study.^16^ The following criteria were satisfied by every single SNP that was requested: 1. There was a substantial correlation with the amount of exposure based on the relevance of the genome as a whole; 2. No linkage disequilibrium (LD) (pairwise r^2^ = 0.001, window size = 10,000kb); 3. Not containing any palindromic structures. A total of 65 SNPs were found after considering the above three assumptions and criteria. We were unable to find the appropriate SNPs in the atherosclerosis GWAS, thus to get accurate estimates, we employed proxy SNPs that had substantial LD (r^2^>0.8) to stand in for the chosen SNPs, which allowed us to get more accurate results. The first-stage regression, or F statistic, was used to assess the strength of the instruments and was calculated using the following equation: F= (R^2^/k)/ ([1−R^2^]/[n−k−1]), where R^2^ is the proportion of the sleep duration variability accounted for by the SNP, k is the number of instruments used in the model and n is the sample size.^20^ To limit the influence of possible weak IV bias, an F statistic greater than 10 was expected to be of sufficient strength for the main study.^21^ The flow chart for the selection of IVs is depicted in Figure 1.

**Figure 1:** The flow chart of instrumental variables selection.

### Statistical analysis

The inverse-variance weighted (IVW) approach was used as the main method to determine whether there was a correlation between sleep duration and atherosclerosis.^22^ If the p from Cochran's Q test was greater than 0.05, we decided to use a model with fixed effects; in all other cases, we used a model with random effects.^23^ If the selected IVs did not exhibit directional pleiotropy (and the *p* for the MR-Egger intercept was greater than 0.05), the IVW technique was considered the most reliable.^24^

We chose the MR-Egger approach to assess the possible pleiotropy impacts in sensitivity analyses. The MR-Egger regression's intercept term, which estimated the causal effect as the slope from the weighted regression of the IVs-outcome relationships on the IVs-exposure relationship, reflected the average pleiotropic effect.^25,26^ To determine whether there was pleiotropy, we also used the basic median, weighted median, Radial MR, and MR-PRESSO (Mendelian Randomization Pleiotropy Residual Sum and Outlier) outlier test techniques.^26^ If more than fifty percent of the SNPs being studied were effective IVs, then the weighted median will offer the most reliable estimates of the causative impact. In addition to pleiotropy detection, MR-PRESSO also can reevaluate effect estimations and eliminate outlier SNPs.^26^ To evaluate the impact of outlying data, a leave-one-out analysis was conducted in the meantime. We further investigated each chosen SNP's pleiotropy using the PhenoScanner V2 database (http://www. pen scanner. medschl. cam. ac. uk/) at the GWAS level of statistical significance (p < 5×10^−8^) to exclude the impact of other variables.^27^

Unless otherwise stated, all tests were two-sided, and the differences were regarded as statistically significant (p < 0.05). The R software's Two Sample MR (V 0.5.6), Radial MR, and MR-PRESSO (V 1.0)^24^ packages were used for all statistical analyses (4.0.5).

## Results

Further information on the chosen SNPs is given in Supplementary Table S1-S2. Three SNPs in total (sleep duration: rs1611719, rs17732997, and rs2186122) were eliminated from the MR research because they were palindromes. In the end, 62 SNPs, including 1 proxy SNP, were chosen as IVs (all p < 5×10^−8^, r^2^=0.001).

### MR estimates

The results of Cochran's Q test for sleep time showed minimal heterogeneity (p = 0.108). According to the IVW method's findings, there was little evidence of a link between the length of sleep and the risk of atherosclerosis (OR (95% CI), 0.992 (0.979-1.004); p = 0.186) (Supplementary Table S3).

### Sensitivity analyses

The results of the simple median and weighted median were comparable to those of the IVW approach. Meanwhile, horizontal pleiotropy was not detected by the MR-Egger regression (p intercept=0. 071 for sleep duration) (Supplementary Table S3). Although Radial MR suggested the existence of outliers (Figure 2), MR-PRESSO showed that outliers did not affect the study results (Table 1). Similarly, the pleiotropy was not detected by RAPS (Table 2) and PhenoScanner V2 database. When the horizontal pleiotropy showed p>0.05, IVW (fixed effects) method (Table 2) was used to assess the data. For sleep duration, Supplementary Figures S1-S4 present forest plots, scatter plots, funnel plots, and MR leave-one-out plots.

**Figure 2:** An overview of the radial MR estimates outlier.

**Table 1:** MR-PRESSO estimates between sleep duration and atherosclerosis

Caracteristic	Raw estimate				Outlier corrected estimate			
	N	OR	95%CI	p	N	OR	95%CI	p
Sleep duration	62	0.991	0.979-1.003	0.163	62	0.991	0.979-1.003	0.163

* SNP: single-nucleotide polymorphism; MR-PRESSO: Mendelian randomization pleiotropy residual sum and outlier test. OR: odds ratio; 95%CI: 95% confidence interval.

**Table 2:** RAPS estimates and IVW (fixed effects) between sleep duration and atherosclerosis

Caracteristic	RAPS				IVW (fixed effects)			
	N	OR	95%CI	p	N	OR	95%CI	p
Sleep duration	62	0.992	0.980-1.004	0.193	62	0.992	0.980-1.004	0.135

* SNP: single-nucleotide polymorphism; RAPS: Robust adjusted profile score; IVW (fixed effects). Inverse variance weighted (fixed effects). OR: odds ratio; CI: confidence interval.

### Bias and power analyses

The bias of the genetic instruments was 0.000 for sleep duration. The F statistic of the selected SNPs was 15.671, which was expected to have sufficient strength for the main study (Supplementary Table S3). The estimate derived from the ratio technique was close to the conditional Odds ratio under certain particular conditions and approximated a population-averaged odds ratio.^28,29^ The consistency of the estimator under the null was unaffected by the odds ratio estimate that was used. We performed the power calculations, and the Type 1 error value for sleep duration was 0.05. For the statistical power value for sleep, the duration was 95%. According to the sample size used in the atherosclerosis GWAS meta-analysis, there was >80% power to identify the relationship between the amount of sleep and the risk of atherosclerosis for effect size (OR) of 0.992 (Supplementary Table S4). In recent additional MR investigations, all IVs for genetically predicted sleep duration have been authorized and used.^30,31^ In addition to this, none of them had any bearing on high blood pressure or elevated levels of low-density lipoprotein (Supplementary Table S3).

## Discussion

In this present study, we attempted to explore the causal association between sleep duration and atherosclerosis using an MR method. Our findings showed no evidence that genetically predicted sleep duration is linked to the risk of atherosclerosis in European populations. Additionally, sensitivity studies showed that the findings were generally reliable.

Coronary artery calcium scores (CACS), CIMT, and Brachial-Ankle Pulse Wave Velocity (baPWV) were major surrogate indicators of atherosclerosis and predictors of cardiovascular events.^6,32^ Some studies have explored the effect of sleep duration on the incidence of atherosclerosis by analyzing the relationship between sleep duration and CACS, CIMT, and baPWV. A recent study of 1,968 healthy men aged 40 to 60 indicated that increased or decreased sleep duration was associated with an increased incidence of coronary atherosclerosis and assessed the effect of sleep duration on the incidence of subclinical arteriosclerosis by measuring CACS, finding people who slept for 7 hours had the lowest incidence of subclinical coronary atherosclerosis.^33^ For people with risk factors for atherosclerosis, sleep duration was also significantly correlated with the incidence of atherosclerosis. Similarly, the CIMT was the lowest when the sleep time was 7-8 h, and the increase or decrease in sleep time will lead to an increase of CIMT.^12^

Previous studies showed no relationship between sleep duration and markers of vascular damage and atherosclerosis.^9,34-38^ A cross-sectional survey of 1,093 Japanese men reported that self-reported sleep duration was not associated with increased CAC or CIMT.^33^ Souza et al.^35^ also found no independent associations of objective sleep duration with CIM. No evidence demonstrated that the association between insomnia symptoms and CAC score >0 differed by objective short sleep duration status.^36^ In addition, a study on the Multi-Ethnic Study of Atherosclerosis (MESA) showed that severe obstructive sleep apnea was not associated with high CAC burden or abnormal ABI.^37^ MESA investigators reported no associations between short (<6 hours) and long (>8 hours) sleep durations and CAC.^38^ These were consistent with our findings.

The clinically and physiologically significant link between extended sleep and CVDs risk in adults is not supported by sufficient experimental data. We hypothesized, based on the current information, that the underlying mechanism was metabolic in nature and operated through an inflammatory route. Specifically, prolonged sleep may lead to low HDL,^39,40^ hyperglycemia, hypertriglyceridemia,^41^ and insulin resistance,^42^ all of which can lead to vascular endothelial dysfunction and subclinical inflammation, thereby further promoting atherosclerosis.^43,44^ Related social, lifestyle, and behavioral issues, such as drug abuse, physical inactivity, or lack of access to nutritious meals, may exacerbate this pro-atherogenic environment.^45,46^ Regardless of the actual cause-and-effect relationship, we supported the examination of sleep length in clinical assessments, as short or long sleep duration may indicate the risk of chronic diseases. CVDs and diabetes are life-threatening diseases that are prevalent in our society and can lead to early illness and death, thus it is important to investigate the relationship between sleep and chronic diseases over time. This includes finding the best prevention strategy to warn against atherosclerosis, which can stimulate CVDs and other diseases. This is an important step towards a healthier population both nationally and globally.

According to our knowledge, this study was the first MR investigation to examine the causal association between sleep duration and risk of atherosclerosis using available GWAS datasets. Additionally, for this two-sample MR investigation, we chose people from Europe to lessen demographic bias. The current MR research also had several shortcomings. First, since we used publicly accessible genetic data for our investigation, we were unable to do stratified analysis or take additional factors into account. Second, the chosen instrumental SNPs as IVs only partially (0.001%-0.01%) explained the variation in sleep duration. Low statistical power to identify weak relationships may result from this. Third, in a two-sample MR analysis, any bias due to weak instruments was in the direction of the null. Bias in the direction of the null was less serious than bias in the direction of the observational association, as it is conservative and will not lead to inflated Type 1 error rates and false-positive findings. There was indeed a possibility of overlap between the two samples.^28^ Ultimately, since our data set was made up of people of European heritage, our conclusions may not apply to other groups outside of Europe.

## Conclusions

In the current study, genetically predicted sleep duration among European populations was not causally linked to the risk of atherosclerosis. Further study is needed to investigate the causal association between atherosclerosis and sleep duration.

## *Supplemental Materials

For additional information Supplemental Table, please click here.

For additional information Supplemental Figure, please click here.
